# Microarray based comparative genome hybridization detects genomic imbalances deletions and duplications in different epileptic patients of Saudi Arabia

**DOI:** 10.1186/1471-2164-15-S2-P49

**Published:** 2014-04-02

**Authors:** Muhammad Imran Naseer, Muhammad Faheem, Maha Mohsin Al-Quaiti, Adeel G Chaudhary, Nobutaka Hirokawa, Mohammed H Al-Qahtani

**Affiliations:** 1 Center of Excellence In Genomic Medicine and Research (CEGMR), King Abdulaziz University 21589, Jeddah, Saudi Arabia; 2 Department of Biochemistry, Faculty of Science, King Abdulaziz University, Jeddah, Saudi Arabia; 3 Department of Cell Biology and Anatomy, and Molecular Structure and Dynamics, University of Tokyo, Japan

## Background

Epilepsy is genetically complex neurological disorder affecting millions of people of different age groups varying in its type and severity. Copy number variants (CNVs) are the key players in the genetic etiology of numerous neurodevelopmental disorders and prior findings also revealed that chromosomal aberrations are more susceptible against the pathogenesis of epilepsy [1].

## Materials and methods

We performed genome wide study of CNVs in epileptic patients by using high density array comparative genome hybridization (CGH) technology for the identification of novel chromosomal aberrations. For this purpose, a cohort of 60 patients suffering with epileptic disorders was recruited. Investigation by array CGH was done for the detection of their chromosomal aberrations. The attained results were analyzed by microarray data analysis software PARTEK and the novelty of CNVs was checked by using Database of Genomic Variants (DGV). Furthermore, array CGH results amplification and deletions were confirmed by quantitative real time PCR.

## Results

Our results showed CNVs including the amplifications, deletions and amplifications plus deletions in different chromosomal regions in the patients. Amplifications were observed in the chromosomal regions 1p21.3, 2p21, 5p14.3, 5q23.2, 6p12.1, 7p15.2, 19p13.13 respectively whereas the deletions were observed in the chromosomal regions 5p14.3, 7q32.3, 19p13.13 respectively. Amplifications plus deletions were observed in 5p14.3, 19p13.13. Moreover, the array CGH results were also validated by quantitative real time PCR.

## Conclusions

Finally, we found some of novel genes in our study for the first time in Saudi population. Further analysis of the observed deleted and duplicated genes by array CGH were confirmed by using quantitative real time PCR. Hence, it is recommended that array CGH as well as quantitative real time PCR can be used for screening of novel epileptic genes. These advanced approaches used in this study for the first time aims to identify novel mechanisms underlying epileptic disorder, which can help to improve the clinical management of individual cases in lowering the burden of epilepsy in Saudi Arabia.
